# The vitamin D receptor and inducible nitric oxide synthase associated pathways in acquired resistance to *Cooperia oncophora *infection in cattle

**DOI:** 10.1186/1297-9716-42-48

**Published:** 2011-03-17

**Authors:** Robert W Li, Congjun Li, Louis C Gasbarre

**Affiliations:** 1 Animal and Natural Resources Institute, United States Department of Agriculture, Agricultural Research Service, Beltsville, MD 20705, USA

## Abstract

*Cooperia oncophora *is an economically important gastrointestinal nematode in ruminants. Acquired resistance to *Cooperia oncophora *infection in cattle develops rapidly as a result of prior infections. Naïve cattle, when given a primary infection of high-dose infective L3 larvae, develop a strong immunity to subsequent reinfection. Compared to primary infection, reinfection resulted in a marked reduction in worm establishment. In order to understand molecular mechanisms underlying the development of acquired resistance, we characterized the transcriptomic responses of the bovine small intestine to a primary infection and reinfection. A total of 23 pathways were significantly impacted during infection. The vitamin D receptor activation was strongly induced only during reinfection, suggesting that this pathway may play an important role in the development of acquired resistance via its potential roles in immune regulation and intestinal mucosal integrity maintenance. The expression of inducible nitric oxide synthase (NOS2) was strongly induced during reinfection but not during primary infection. As a result, several canonical pathways associated with NOS2 were impacted. The genes involved in eicosanoid synthesis, including prostaglandin synthase 2 (PTGS2 or COX2), remained largely unchanged during infection. The rapid development of acquired resistance may help explain the lack of relative pathogenicity by *Cooperia oncophora *infection in cattle. Our findings facilitate the understanding of molecular mechanisms underlying the development of acquired resistance, which could have an important implication in vaccine design.

## Introduction

*Cooperia oncophora *is one of the most economically important gastrointestinal nematodes in ruminants that result in production inefficiency. In Brazil, over 70% of parasites recovered from cattle grazed on pasture belong to the genus *Cooperia *[1]. While clinical symptoms are generally absent or relatively mild, *C. oncophora *infection has been shown to reduce live weight gain as much as 13.5% of total cattle bodyweights [2], possibly due to inappetence and nutritional deficiency. Pathophysiological changes induced by infection are typically restricted to the site of infection, mainly in the duodenum and jejunum. These changes include morphological and structural alterations in intestinal villi [2,3], loss of plasma protein into the gut [3] and enhanced mucus excretion [3,4].

*C. oncophora *infection in cattle elicits a Th2-like immune response, characterized by up-regulation of IL-4 and the involvement of both eosinophils and mucosal IgA [4-7]. Host serological response to *C. oncophora *infection has been extensively studied [6,8-10]. *C. oncophora*-specific serum and mucosal IgG1 and IgA are strongly induced upon experimental challenge in cattle [8]. Moreover, the levels of *Cooperia*-specific IgA are significantly higher in intermediate responders than in low responders in cattle [9] and expulsion of the adult *Cooperia *worm appeared to be associated with a significant increase in mucosal IgA and an influx of eosinophils [6]. PIGR, a gene responsible for trans-epithelial transport of polymeric immunoglobulins, such as IgA dimers and IgM pentamers, into mucosal and glandular secretions, is strongly up-regulated in the heifers resistant to parasitic nematodes after experimental parasite challenge [7]. The peak in antibody titres is preceded by a significant increase in B and MHCII^+ ^cells in the draining lymph nodes, suggesting that B cells may play an important role in development of acquired immunity against the parasite [8].

In ruminants, adult animals often exhibit acquired resistance to gastrointestinal nematodes. This process tends to display a temporal characteristic. The ability to reject incoming larvae is first acquired, followed by depressing female fecundity and then by expelling adult worms by the host. The prevention of worm establishment in the host tissue, which is determined by host age, seems most important and requires a threshold worm burden in order to invoke a strong immune response [11]. Compared to the abomasal nematode *Ostertagia ostertagi*, which requires a prolonged exposure before acquired resistance becomes effective [12,13], protective immunity to reinfection in cattle develops rapidly following a primary *C. oncophora *infection. A significant reduction in worm burdens can be achieved in primed cattle with a primary infection [14]. In these animals, acquired resistance is also manifested by increased percentage of L4 larvae. In addition, worm length and fecundity are also significantly reduced. This observed immunity is also confirmed in cattle during natural infection. Calves moderately exposed to *C. oncophora *during the first grazing season are absent of any *C. oncophora *larvae in their fecal cultures during the second grazing season [15,16]. Accumulated evidence suggests that a rapid development of protective immunity may well explain relatively mild clinical symptoms and lack of severe pathogenicity observed in cattle exposed to *C. oncophora *infections. While the effect of host response types or genetic factors on worm establishment and infection doses on worm morphology and reproduction are well understood, molecular mechanisms underlying the development of acquired resistance in cattle have not received any attention. In this study, we aim to understand the underlying mechanisms that contribute to the development of acquired resistance against *C. oncophora *infection in cattle, which should have a positive impact on formulating optimal drug-independent nematode control strategies.

## Materials and methods

### Animals and worm burdens

Sixteen Holstein bull calves were purchased locally within 48 h after birth. The animals were fed *ad libitum *with a standard calf ration and maintained on concrete for the duration of the experiment. These animals were randomly divided into 4 groups (naïve control, primary infection, drug-treated control, reinfection) with 4 animals in each group. After reaching ~ 4 months of age, twelve of these 16 nematode-naïve animals were orally infected with a single dose of *C. oncophora *infective L3 larvae (10^5 ^larvae per animal) for 14 days post infection (dpi). The L3 larvae were obtained from cultures maintained at the USDA-ARS Beltsville facilities. Four uninfected naïve animals were used as controls. Four out of the 12 infected animals (primary infection) at 14 dpi and four naïve control animals were sacrificed. The remaining 8 infected calves were treated with a 2× labeled dose of fenbendazole (Safe-Guard) to remove existing parasites. These calves were allowed to rest for 30 days on concrete. Four of the 8 drug-treated calves were then infected with a single dose of 10^5 ^L3 larvae per animal for 14 days (reinfection) and the remaining four drug-treated calves remained uninfected and served as drug-treated controls. Small intestine tissues were collected from the jejunum approximately three meters from the pyloric sphincter and snap frozen in liquid nitrogen prior to storage at - 80°C until total RNA was extracted. The animal maintenance and handling were based on the protocol approved by The USDA-ARS Animal Care and Use Committee; and Institutional Animal Care and Use Committees guidelines were strictly followed. Fecal egg count (egg per gram of feces or EPG) was monitored during the repeat infection experiment using zinc sulfate double centrifugation and parasite burdens were determined as previously described [13].

### RNA Extraction, real-time RT-PCR, and microarray analysis

Total RNA extraction, real-time RT-PCR and microarray fabrication and hybridization were performed as previously described [7,17]. Briefly, total RNA was extracted using Trizol (Invitrogen, Carlsbad, CA, USA) and further purified using an RNeasy Mini kit (Qiagen, Valenica, CA, USA). RNA integrity was verified using a Bioanalyzer 2100 (Agilent, Palo Alto, CA, USA). For real-time RT-PCR, the cDNA synthesis was performed with an iScript cDNA Synthesis kit (Bio-Rad, Hercules, CA, USA). Real-time RT-PCR analysis was carried out with the iQ SYBR Green Supermix kit (Biorad) using 200 nM of each amplification primer and the 1^st^-strand cDNA (100 ng of the input total RNA equivalents) in a 25 μL reaction volume as described [7]. The amplification was carried out on an iCycler iQ™ Real Time PCR Detection System (BioRad) with the following profile: 95°C-60 s; 40 cycles of 94°C-15 s, 60°C-30 s, and 72°C-30 s. A melting curve analysis was performed for each primer pair. The ribosomal protein S29 (RPS29), with relatively constant expression levels across all experiment samples, was used as an endogenous control. Relative gene expression data was calculated using the 2^-ΔΔCT ^method [18]. The fold change was normalized against the naive group.

The bovine microarray, which included 86 191 unique 60mer oligonucleotides synthesized in situ, each repeated 4 times on the microarray, representing 45 383 bovine genes and/or expressed sequence tags (ESTs), was previously described [17]. After hybridization, scanning and image acquisition, data were extracted from raw images using NimbleScan software (Roche, Indianapolis, IN, USA). A total of 16 microarrays, 4 biological replicates per treatment group, were used in this experiment (GEO accession# GSE24402). Relative signal intensities (log2) for each feature were generated using the robust multi-array average algorithm [19]. The data were then processed based on the quantile normalization method [20]. The background-adjusted, normalized, and log-transformed intensity values were further analyzed using MeV v4.2. [21]. Significantly regulated genes were identified using the method suggested by Guo et al. [22] based on their significance (*P *< 0.05) and followed by fold change (2 fold as a cutoff). A nucleotide BLAST was conducted against the Reference mRNA Sequences Database (RefSeq) for all sequences that were significantly impacted during infection using 60mer oligo sequences on the microarray (cutoff E Value < 10^-8^). These genes are listed in Additional file 1. After removing redundancy in which a gene was represented by multiple sequences, genes with annotation and approved gene symbols (Table 1) were used for pathway analysis discussed below.

**Table 1:** Genes significantly induced in the bovine small intestine by *Cooperia oncophora *infections*.

Bovine RefSeq_ID	Symbol	Description	Primary infection	Reinfection
				
XM_587930.4	ABCG1	similar to ATP-binding cassette sub-family G member 1	2.29*	1.08
NM_174501.2	ALOX15	arachidonate 15-lipoxygenase	1.27	2.42*
NM_001083508.1	BAALC	brain and acute leukemia, cytoplasmic	0.51	2.07*
XM_587691.3	BEST4	similar to bestrophin 4	0.74	2.05*
XM_876130.3	BZW1	basic leucine zipper and W2 domains 1	2.78*	1.33
NM_001104961.1	BZW2	basic leucine zipper and W2 domains 2	3.89*	0.66
NM_001076195.1	CALB1	calbindin 1, 28 kDa	1.15	2.13*
NM_001017934.2	CCT3	chaperonin containing TCP1, subunit 3 (gamma)	2.06*	0.52
XM_869285.3	CDH26	similar to cadherin-like 26	39.48***	13.08*
XM_583707.4	CDK8	similar to MGC81962 protein	2.55*	1.26
NM_176788.1	CEBPB	CCAAT/enhancer binding protein (C/EBP), beta	1.15	2.09*
XM_001790249.1	CKAP5	similar to colonic and hepatic tumor over-expressed protein	2.20*	0.81
NM_001101980.1	COL17A1	collagen, type XVII, alpha 1	0.76	2.06*
NM_001034340.1	CYR61	cysteine-rich, angiogenic inducer, 61	0.87	2.62**
NM_001102301.1	DDN	dendrin	2.35*	0.97
NM_001046036.1	DIMT1L	DIM1 dimethyladenosine transferase 1-like (S. cerevisiae)	2.77*	1.23
NM_001010992.3	DIO2	deiodinase, iodothyronine, type	4.38*	2.41*
NM_001046052.1	DYNC1LI1	dynein, cytoplasmic 1, light intermediate chain 1	2.09*	0.74
NM_001143864.1	ELP2	elongation protein 2 homolog (S. cerevisiae)	3.82**	1.11
NM_001102519.1	ENDOD1	endonuclease domain containing 1	1.45	2.49*
XM_614853.4	ETV5	similar to ets variant gene 5 (ets-related molecule)	2.53	2.32*
NM_001008665.1	F11	coagulation factor XI	1.01	2.01*
NM_174541.2	GABRA2	gamma-aminobutyric acid (GABA) A receptor, alpha 2	1.57	2.34**
NM_001075844.1	GABRA5	gamma-aminobutyric acid (GABA) A receptor, alpha 5	2.34	0.49**
NM_001038143.1	GCLM	glutamate-cysteine ligase, modifier subunit	1.22	2.50**
NM_205809.1	GCNT3	glucosaminyl (N-acetyl) transferase 3, mucin type	7.66**	2.47
XM_868229.3	GIF	similar to gastric intrinsic factor (vitamin B synthesis)	0.80	3.69**
XM_865266.3	GPR120	similar to G protein-coupled receptor 120	2.02*	1.25
NM_001080354.1	GSTM4	glutathione S-transferase mu 4	2.81*	1.61
XM_605913.4	HERC6	similar to hect domain and RLD 6	0.74	2.62*
NM_001031751.1	HGF	hepatocyte growth factor	2.05*	1.43
NM_001105651.1	HLA-A	major histocompatibility complex, class I, A	2.02*	0.43
XM_001253142.1	KSR2	similar to Kinase suppressor of Ras 2	2.10	0.50*
NM_001076122.1	HS3ST1	heparan sulfate (glucosamine) 3-O-sulfotransferase 1	1.22	2.00**
NM_001040563.1	HTATIP2	HIV-1 Tat interactive protein 2, 30 kDa	2.07*	1.15
NM_174644.2	IDH3A	isocitrate dehydrogenase 3 (NAD+) alpha	2.27*	1.24
NM_001075588.1	IFI6	interferon, alpha-inducible protein 6	0.17	2.55*
NM_001046210.1	IL1R2	interleukin 1 receptor, type II	1.03	2.06*
XM_590057.4	KIAA0415	hypothetical protein LOC512522	1.30	2.45*
NM_001046411.1	KRT7	keratin 7	2.30*	1.87
XM_595458.4	LIPM	similar to Lipase member M precursor	1.57	2.21*
XM_001249810.2	LONRF3	similar to LON peptidase N-terminal domain and RING finger protein 3	0.97	2.06*
NM_173933.2	LPO	lactoperoxidase	2.33	0.16**
NM_001097565.1	LRP8	low density lipoprotein receptor-related protein 8	2.57*	1.79
XM_001789987.1	METTL8	methyltransferase like 8	0.87	2.20*
NM_173940.2	MX1	myxovirus (influenza virus) resistance 1	0.54	2.05*
XM_613028.3	NEB	nebulin	1.82**	2.25*
NM_001076799.1	NOS2	nitric oxide synthase 2, inducible	0.97	2.18**
XM_592814.2	P2RX1	similar to P2X purinoceptor 1 (ATP receptor)	3.14*	1.19
NM_001001600.1	PGA5	pepsinogen 5, group I (pepsinogen A)	1.21	2.20*
XM_583514.4	PGM2	phosphoglucomutase 2	2.89*	0.70
NM_001035017.1	PHGDH	phosphoglycerate dehydrogenase	2.62*	1.09
XM_601308.3	POLS	similar to DNA polymerase sigma	1.01	2.06**
NM_174432.2	PRDX3	peroxiredoxin 3 (PRDX3)	2.16*	0.51
NM_174690.1	PRSS2	protease, serine, 2 (trypsin 2)	3.44*	1.13
NM_001105323.1	PTGS1	prostaglandin-endoperoxide synthase 1	2.39*	1.52
NM_001103316.1	PTPLAD1	protein tyrosine phosphatase-like A domain containing 1	1.45	2.40*
NM_001046303.1	RELL2	RELT-like 2	2.07	0.27**
NM_001080232.1	RGS13	regulator of G-protein signaling 13	1.26	2.63*
NM_001045941.1	RSAD2	radical S-adenosyl methionine domain containing 2	0.40	3.18*
NM_173959.4	SCD	stearoyl-CoA desaturase (delta-9-desaturase)	2.21*	0.95
XM_590757.3	SEMA3B	Sema domain, short basic domain, secreted,3B	0.77	2.03*
NM_001099211.1	SF3A2	splicing factor 3a, subunit 2, 66 kDa	1.03	2.12*
NM_001099378.1	SLC15A1	solute carrier family 15 (oligopeptide transporter), member 1	2.19*	0.57
XM_867835.3	SLC22A15	similar to solute carrier family 22, member 15	1.58	2.57*
NM_176640.2	SLC35A2	solute carrier family 35 (UDP-galactose transporter), member A2	2.07*	1.30
XM_001790621.1	SLC38A1	solute carrier family 38, member 1	2.50*	1.70*
NM_001101994.1	SLC6A12	solute carrier family 6, member 12	2.08	0.32*
NM_174187.2	SPP1	secreted phosphoprotein 1	1.01	4.69*
NM_001046456.1	TICAM2	toll-like receptor adaptor molecule 2	3.29*	1.04
NM_001035107.1	TINAG	tubulointerstitial nephritis antigen	1.69	3.41*
NM_001076856.1	TMEM66	transmembrane protein 66	2.99*	1.03
XM_600015.4	TNFSF9	similar to tumor necrosis factor (ligand) superfamily, member 9	2.61**	1.02
NM_001038155.1	TNS4	tensin 4	2.84*	1.88
NM_001012284.1	UBA7	ubiquitin-like modifier activating enzyme 7	0.38	2.37*
NM_001103233.1	ULBP3	UL16 binding protein 3	2.23*	0.31
NM_001035075.1	XAF1	XIAP associated factor 1	0.55	2.20**
NM_001102354.1	XRCC2	X-ray repair complementing defective repair in Chinese hamster cells 2	1.40	2.36**
XM_585095.4	ZBP1	similar to Z-DNA binding protein 1	0.44	2.20**
XM_874604.3	ZNF71	similar to zinc finger protein 71	2.98*	1.14
				

*Only sequences with annotation are listed.

Genes significantly regulated during infection were analyzed using Ingenuity Pathways Analysis (IPA) software v8.7 (Ingenuity Systems, Redwood City, CA, USA) as described previously [13,23]. Genes significantly up-regulated and down-regulated were analyzed separately. Genes with known gene identifiers (gene symbols) and their corresponding expression values were uploaded into the software. Canonical pathways were identified based on two parameters: (1) A ratio of the number of genes from the data set that map to the pathway divided by the total number of genes that map to the canonical pathway, and (2) a P value calculated using Fischer's exact test, which determines the probability that the association between the genes in the data set and the canonical pathway is due to chance alone.

### Western blot analysis

Western blot analysis was essentially the same as described [13]. Briefly, crude proteins were extracted from bovine small intestine samples using Mammalian Protein Extraction Reagent (Pierce, Rockford, IL, USA) with a protease inhibitor cocktail (Sigma, St. Louis, MO, USA) added prior to use. After homogenization, the samples were briefly centrifuged at 4°C for 2 min at 10 000 × *g *to remove debris. Crude protein was quantified using a modified Branford method and Western blot analysis performed as described [4]. Briefly, the protein from different samples was separated by SDS PAGE on 2 identical 4 to 20% polyacrylamide gradient gels. One gel was stained with Simply Blue Safestain (Invitrogen) and served as loading control. Another gel was used for Western blot and imaging analysis. The Western blot was probed with the following primary antibodies, SPP1 (OPN) mouse monoclonal antibody (sc-21742, Santa Cruz Biotechnology, Santa Cruz, CA, USA) and GCNT3 goat polyclonal antibody (ab77728, Abcam, Cambridge, MA, USA). After washing, these blots were incubated with an IRdye labeled secondary antibody (Li-Cor Bioscience, Lincoln, NE, USA). The bands were detected using a Li-Cor Odyssey Infrared Imaging System (Li-Cor). The relative density of the target bands on the blots was quantified using the imaging software UN-SCAN-IT (Silk Scientific, Orem, UT, USA).

## Results

### Parasitology and worm counts

The naïve control calves remained worm-free during the experiment. No worms were recovered from drug-treated control animals, suggesting that a 2× labeled dose of fenbendazole (Safe-Guard) is sufficient to eliminate existing parasites. EPG was monitored weekly during the rest period. A low EPG value (EPG = 8) was detected only in one of the animals in a single time point, providing further evidence the efficacy of drug drenching. Approximately a 56% reduction in worm burden was observed in reinfected calves (mean worm counts ± SD = 10 334 ± 3 585) compared to the animals with only a primary infection (23 883 ± 7 833) at 14 dpi). The difference is marginally significant (*P *< 0.1), probably due to a substantial variation in worm burden in an out-bred population used in this challenge experiment. While uncharacterized genetic makeup of experiment animals and a small sample size used in challenge studies are a major concern, a significant reduction in worm burden and a higher percentage of immature larvae recovered in reinfected animals (15.2% vs. 9.4% in primarily infected animals, *P *< 0.1) suggested these animals had indeed acquired protective immunity to infection.

### Transcriptomic profiles and pathway analysis

Transcriptomic disruptions in the bovine jejunum induced by *C. oncophora *between a primary infection and a drug-attenuated reinfection were compared using a bovine high-density microarray consisting of 86 191 unique 60mer oligonucleotides. Based on both significance derived from an unpaired *t*-test (*P *≤ 0.05) and fold change, a total of 308 unique sequences were impacted during infection. The alteration in the bovine transcriptome by the parasite infection appeared to be minimal; and only a small fraction of the transcriptome (< 1%) was affected. Among the 308 sequences induced, eighty unique genes that were significantly up-regulated can be identified with annotation (Table 1).

Approximately forty genes were significantly up-regulated during the primary infection only (Table 1). These genes include basic leucine zipper and W2 domains 1 and 2 (BZW1 and BZW2), dendrin (DDN), glucosaminyl (N-acetyl) transferase 3, mucin type (GCNT3), hepatocyte growth factor (HGF), major histocompatibility complex, class I, A (HLA-A), toll-like receptor adaptor molecule 2 (TICAM2), and UL16 binding protein 3 (ULBP3). On the other hand, at least 44 genes, such as calbindin 1 (CALB1), collagen, type XVII, alpha 1(COL17A1), interferon, alpha-inducible protein 6 (IFI6), interleukin 1 receptor, type II (IL1R2), nitric oxide synthase 2, inducible (NOS2), and secreted phosphoprotein 1 (SPP1, osteopontin), were significantly induced only during reinfection. Several genes, such as cadherin-like molecule 26 (CDH26), nebulin (NEB), deiodinase, iodothyronine, type II (DIO2), and solute carrier family 38, member 1 (SLC38A1), were significantly up-regulated during both the primary infection and reinfection. Genes significantly repressed during infection, such as adrenergic, alpha-1B-, receptor (ADRA1B), cytochrome P450, family 4, subfamily A, polypeptide 11 (CYP4A11), and hypoxia inducible factor 3, alpha subunit (HIF3A), were listed in Additional file 1.

**Figure 1:**
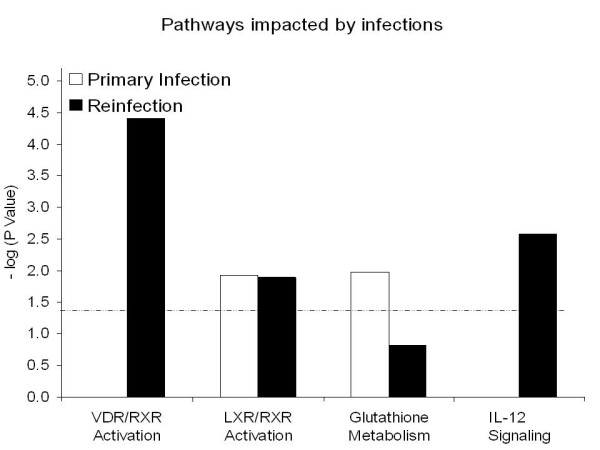
**Selected pathways significantly impacted in the bovine small intestine during *Cooperia oncophora *infection at 14 dpi**. The dashed line represents a significance level at *P *< 0.05. *N *= 4.

Twenty three pathways were significantly impacted (*P *< 0.05) during infection (Additional file 2). Eight pathways in which their genes were down-regulated during primary infection included fatty acid metabolism, lysine degradation, fatty acid elongation in mitochondria, and LPS/IL-1 mediated inhibition of RXR function. During reinfection, three pathways whose genes were down-regulated, such as calcium signaling and fatty acid metabolism, were significantly impacted. Fatty acid metabolism was seemingly the only pathway suppressed during both primary infection and reinfection. Twelve pathways were significantly stimulated during infections as evidenced by up-regulation of the genes involved in these pathways. LXR/RXR activation was the only pathway stimulated during both primary infection and reinfection while the VDR/RXR activation pathway was stimulated only during reinfection (Figure 1). Notably, NOS2 was involved in 5 out the 8 pathways impacted during reinfection. The eicosanoid pathway, which is significantly impacted only at 28 dpi during *C. oncophora *primary infection in cattle [7], was not seemingly involved in the development of acquired resistance.

### Quantitative PCR and Western blot analysis

**Figure 2:**
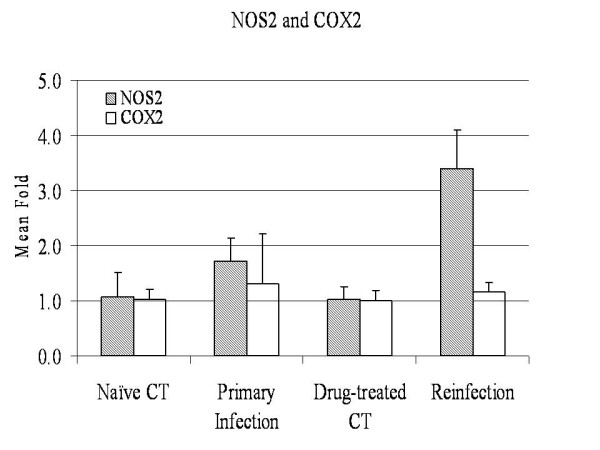
**The expression profiles of NOS2 and COX2 in the bovine small intestine during *Cooperia oncophora *infection at 14 dpi**. The expression value at the mRNA level was detected using quantitative RT-PCR. The expression value of the naïve control group was set as 1.0. The fold change (*N *= 4) were calculated using the 2^-ΔΔCT ^method and normalized against the naïve control group (mean ± SD). NOS 2 = nitric oxide synthase 2, inducible; COX2 = PTGS2 (prostaglandin-endoperoxide synthase 2 (prostaglandin G/H synthase and cyclooxygenase)).

**Figure 3:**
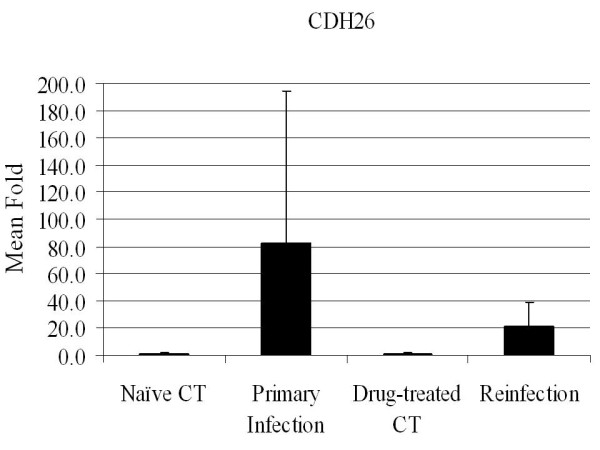
**CDH26 expression in the bovine small intestine during *Cooperia oncophora *infection**. The expression value at the mRNA level was detected using quantitative RT-PCR. The expression value of the naïve control group was set as 1.0. The fold change (*N *= 4) were calculated using the 2^-ΔΔCT ^method and normalized against the naïve control group (mean ± SD). CDH26 = cadherin 26.

**Figure 4:**
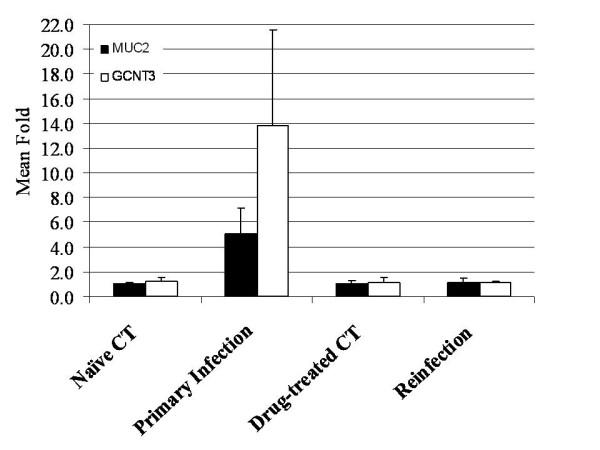
**The expression of two genes in mucin biosynthesis in the bovine small intestine during *Cooperia oncophora *infection at 14 dpi**. The expression value at the mRNA level was detected using quantitative RT-PCR. The expression value of the naïve control group was set as 1.0. The fold change (*N *= 4) were calculated using the 2^-ΔΔCT ^method and normalized against the naïve control group (mean ± SD). MUC2 = mucin 2, oligomeric mucus/gel-forming; GCNT3 = glucosaminyl (N-acetyl) transferase 3, mucin type.

The mRNA expression of 16 genes was analyzed using real-time RT-PCR (Additional file 3), which were selected on the basis of their presumed biological relevance. These include 10 genes in the eicosanoid pathway such as 5-lipoxygenase activating protein (ALOX5AP or FLAP), prostaglandin synthase 2 (PTGS2 or COX2), leukotriene A4 hydrolase (LTA4H), leukotriene C4 synthase (LTC4), and thromboxane A synthase (TBXAS1). While arachidonate 15-lipoxygenase (ALOX15) was slightly up-regulated during reinfection, consistent with the results obtained using the oligo microarray, other genes, including COX2 (Figure 2), remained largely unchanged. Quantitative PCR also confirmed up-regulation of NOS2 at the mRNA level during reinfection (Figure 2). This gene was significantly up-regulated at 14 dpi based on quantitative PCR analysis, which is in agreement with the microarray results. CDH26 expression was induced to a much greater extent during primary infection than during reinfection, 82 vs. 21 fold (Figure 3), compared to their respective controls. The vitamin D3 receptor (VDR) expression was slightly elevated during both primary infection and reinfection (≈ 1.7 fold) compared to its respective controls. The mRNA level of mucin 5B (MUC5b) in the small intestine was very low and unchanged during infection. However, MUC2 expression was up-regulated during primary infection but not during reinfection. Interestingly, GCNT3 mRNA followed the same pattern as MUC2, which were strongly up-regulated only during primary infection, compared to naïve controls. The relative amount of both MUC and GCNT3 mRNA molecules in the reinfected animals was indistinguishable from the drug-treated controls as well as from naïve controls (Figure 4).

**Figure 5:**
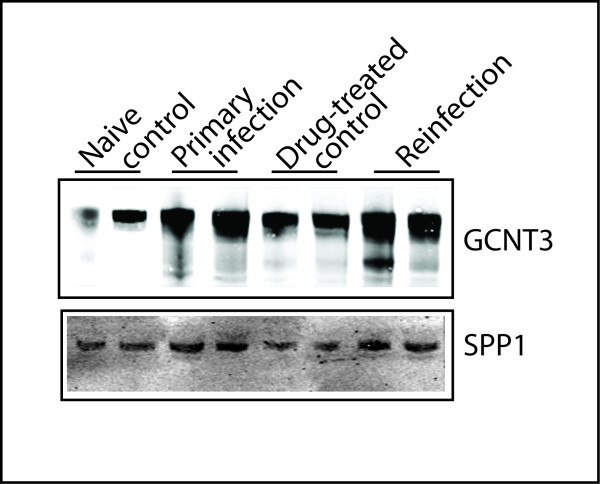
**Western blot analysis of GCNT3 and SPP1 in the small intestine tissue (jejunum) during *Cooperia oncophora *infection at 14 dpi**. The relative densities of the target bands were qualified using UN-SCAN-IT from Silk Scientific. GCNT3 = glucosaminyl (N-acetyl) transferase 3, mucin type. SPP1= secreted phosphoprotein 1 (osteopontin).

Protein expression in the small intestine during infection was monitored using Western blot analysis (Figure 5). SPP1, a gene in the VDR/RXR activation pathway, was two fold higher in the reinfected animals (*N *= 4) compared to the drug-treated control (*N *= 4). This is consistent with mRNA results. SPP1 was also up-regulated in the primed animals (1.8 fold) compared to naïve controls. GCNT3 protein expression was elevated 3.5 fold during primary infection; and its elevated expression was maintained during reinfection but to a lesser extent (2.4 fold) compared to drug-treated controls (Figure 5).

## Discussion

It has long been recognized that genetics plays an important role in the host's ability to resist gastrointestinal nematode infections in ruminants, even though the heritability of the resistance trait is relatively low to moderate in most cases. Different breeds and selection lines in ruminants differ greatly in their relative resistance [24-27]. Resistance is manifested in several distinct forms, including a reduced establishment of worms and retarded or arrested worm development as well as stunting and inhibition of egg production [28]. There have been numerous attempts to exploit the relative contribution of inherited components in susceptibility to GI nematodes in ruminants for its utilities in either applied breeding programs [29] or in understanding molecular mechanisms underlying the trait [17,27]. Acquired resistance, the ability of animals to become resistant after prior infection by pathogens, exposure to stress, or application of chemical inducers, is also well documented. In cattle, calves with previous exposure to a heavily contaminated pasture have a limited establishment of worms, compared to naïve calves that are exposed to the same pasture harboring over 380 times more worms [30]; the ability of calves to acquire resistance to *C. oncophora *appears to be independent of age. Several other studies also support the observation that priming with *C. oncophora *induces strong protective immunity, possibly due to its rapid elicitation of immunological reactions [16]. Understanding genetic and immunological mechanisms underlying the development of acquired resistance could have implications in vaccine design. While temporal responses of cytokine and biochemical pathways to *C. oncophora *infections, both natural and experimental, have been monitored recently [7,31,32], molecular mechanisms underlying the development of acquired resistance have yet to be unraveled.

In this study, twenty three pathways were significantly impacted in the bovine small intestine during *C. oncophora *infection. Among these pathways, the VDR/RXR activation pathway was strongly impacted only during reinfection, suggesting that this pathway may have played an important role in the development of acquired resistance. VDR partners with the retinoid × receptor, RXR, a member of the nuclear hormone receptor family, to form a heterodimer VDR-RXR. The heterodimer is then bound to Vitamin D3 as well as other co-activator proteins to mediate the transcriptional regulation of a number of genes. The activation plays a crucial role in the regulation and metabolism of calcium and phosphorus in the small intestine, kidney and bone as well as modulates the expression of genes in bile acid transport [33]. However, the function of VDR extends far beyond its classical boundary as a regulator of calcium homeostasis and bone metabolism. VDR is constitutively expressed in a variety of immune cells and plays an essential role in gastrointestinal inflammation and innate and adaptive immunity [34]. Mounting evidence suggests any disruption to vitamin D and/or its receptor could have serious consequences in a number of the key physiological processes, including immune function. VDR knock-out (KO) mice exhibit a pro-inflammatory bias and abolish the formation of NF-κB-VDR complex. VDR KO mice have reduced CD4/CD8α α intraepithelial lymphocyte populations in the gut and compromised T cell homing [35]. Therefore, VDR is an important contributor to host protection from bacterial infection and associated with colon tumor progress [36]. Vitamin D3 (calcitriol) treatment in humans induces a significant increase in circulating lymphocytes and the percentage of eosinophil vacuolization [37], a condition favoring a Th2 immune response, a hallmark of parasitic nematode infection. Our results show that increased expression of VDR and strong stimulation of the VDR/RXR activation pathway during *C. oncophora *reinfection may contribute to intestinal repair. Many previously published reports demonstrate that vitamin D3 induces an increased expression of tight junction proteins such as claudins as well as E-cadherin; and its receptor, VDR, and is able to enhance the intercellular junctions [38]. VDR knockdown compromises tight junction functions. VDR plays important roles in maintaining the integrity of the intestinal mucosal barrier. While further evidence is needed to establish a solid link between the VDR pathway and the development of acquired resistance to *C. oncophora *infection in cattle, our findings nevertheless provide a novel direction for future research.

Nitric oxide (NO), one of the most versatile players in the immune system, is critical in host defense because of its cytotoxic and immunoregulatory properties [39,40]. The production of NO by nitric oxide synthases (NOS) in various cell types including macrophages is mainly controlled at the transcriptional level. Inducible nitric oxide synthase (NOS2) is widely expressed in many cell types. NOS2 is readily inducible by cytokines such as TNFα, IL-1β, and IFN- and/or microbial products, resulting in sufficient and sustained production of NO. NO in turn exerts numerous effector and immunoregulatory functions including killing of infectious pathogens and modulating cytokine production and Th cell development [41]. Reactive nitrogen intermediates (NO and its derivatives) are among the key effector molecules of parasite control in the livers of *L. donovani*-infected mice [42]. The host capable of controlling the infection of this intracellular parasite develops an effective T cell- dependent immune response mediated largely by Th1 cytokines, including IL-12 and IFN-γ. On the other hand, Th2 cells play a central role in mediating the protective immunity against parasitic nematode infections by releasing an array of cytokines, such as IL-4 and IL-13. These cytokines, via their receptors such as IL-4 receptor α (IL-4Rα), activates downstream signaling pathways. However, the induction of a Th2-type immune response leading to worm expulsion is complicated. A recent study suggests that neither the expression of this receptor on CD4^+ ^T cells nor macrophages and neutrophils are required for protective immunity to *Trichinella spiralis *infection in mice [43].

*Cooperia oncophora *infection in cattle induces a Th2 immune response. However, dominant effector mechanisms controlling worm expulsion have yet to be identified. A significant increase in mucous IgA and IgG1 as well as an influx of eosinophils are evident during primary infection [7,9]. Th2 cytokines such as IL-4 and IL-13 are strongly up-regulated during a primary infection while TNFα and IFN-γ remains largely unchanged. Results from this study demonstrated that NOS2 expression was up-regulated during reinfection (Figure 2). NOS2 was implicated in 5 of the 8 pathways induced during reinfection, including IL-12 signaling, IL-17 signaling, IL-6 signaling, and glucocorticoid receptor signaling pathways. The expression of IL-12 during *C. oncophora *infection in the bovine small intestine was not monitored in this study. Published studies suggest that ongoing Th2 responses are relatively stable and difficult to switch to a Th1 response [44]. IL-12 is a potent stimulus for Th1 responses and has previously shown to drive chronic *T. muris *infection in a normally resistant mouse strain [45,46]. Resistance can be generated either by a single infection event which exceeds the threshold or multiple sub-threshold infection episodes. The absolute level of parasites required to reach threshold varies between genetically distinct individuals. While resistance is generally associated with Th2 responses, it is possible that the development of acquired resistance to *C. oncophora *infection in cattle requires a delicate balance between the production of Th1 cytokines and Th2 cytokines.

The interaction of pathogen-associated molecular patterns such as carbohydrate moieties on parasites by host pattern recognition receptors (e.g. collectins and galectins) triggers a cascade of events, including activation of various immune cells and subsequent cytokine production and resultant recruitment of leukocytes to the site of infection in the bovine small intestine. A sustained elevation of inflammatory cytokines during priming induces NOS2 gene expression, leading to increased production of NO. These reactive nitrogen species and proteases released by infiltrates create a hostile environment for parasites, which impacts worm establishment and reproduction. Up-regulation of genes in extracellular matrix and tight junction as well as genes involved in mucin biosynthesis by infection may lead to enhanced tissue repair in the small intestine. These factors all contribute to the rapid development of acquired resistance to *C. oncophora *infection in cattle after priming by a high-dose primary infection.

In conclusion, we presented evidence that acquired resistance to *C. oncophora *infection in cattle can be rapidly developed following priming of the immune response. Multiple signaling pathways that were significantly impacted during reinfection were identified, distinct from those during a primary infection. The VDR/RXR activation pathway may have contributed significantly to the development of acquired resistance via its potential roles in immune regulation and intestinal mucosal integrity maintenance. NOS2 expression was strongly induced; and several NOS2 associated pathways were significantly impacted during reinfection, suggesting they may play an important role in protective immunity. However, the development of acquired resistance is likely to be very complicated. The relative contribution of Th1 and Th2 immune responses to the resolution of *C. oncophora *infection in cattle needs to be experimentally defined.

## Competing interests

The authors declare that they have no competing interests.

## Authors' contributions

RWL conceived the study, conducted the experiment, analyzed the data, and drafted the manuscript. CJL carried out Western blot analysis. LCG assisted in parasite infection experiments. All authors read and approved the final manuscript.

## Supplementary Material

Additional file 1 **Genes significantly impacted during *Cooperia oncophora *infections in cattle**. 308 unique sequences significantly regulated during *Cooperia oncophora *infections in cattle. Click here for file

Additional file 2 **Pathways significantly impacted during *Cooperia oncophora *infections in cattle**. 23 canonical pathways significantly impacted during *Cooperia oncophora *infections in cattle. Click here for file

Additional file 3 **Primers used in the experiment**. Primers for 15 genes used in the experiment. Click here for file
